# Supplementary material for: Learning-based multi-objective hyper-heuristic algorithm for reconfigurable assembly line scheduling problems
Source: PLoS One. 2026 May 20;21(5):e0348884. doi: 10.1371/journal.pone.0348884 (PMC13189325; doi:10.1371/journal.pone.0348884)
Supplement: Attachment — Submitted filename: Responses to comments v1.7.docx [file pone.0348884.s001.docx]

**Response to Reviewers**

**Journal:** PLOS ONE

**Submission ID:** PONE-D-26-07514

**Submission Title:** Learning-based multi-objective hyper-heuristic algorithm for reconfigurable assembly line scheduling problems

**Editor-in-Chief**

Thank you for submitting your manuscript to PLOS ONE. After careful consideration, we feel that it has merit but does not fully meet PLOS ONE’s publication criteria as it currently stands. Therefore, we invite you to submit a revised version of the manuscript that addresses the points raised during the review process.

**Response:**

Dear Editor and Reviewers,

We appreciate the time and effort you have invested in helping us improve both the quality and organization of our paper. Each comment raised has been addressed and revisions have been made carefully. Revisions are given in 'blue' colored text in the revised manuscript for easy tracking. Please find below our point-by-point responses to your comments.

Kind regards

Corresponding author

**Academic Editor**

**Comment 1-Academic Editor:**

Clarify the novelty by explicitly distinguishing the proposed approach from existing Q-learning and hyper-heuristic frameworks and prior formulations (e.g., Yuan and Deng, 2017).

**Response 1-Academic Editor:**

We thank the editor for this suggestion. In the revised manuscript, the novelty of the proposed approach relative to existing Q-learning/hyper-heuristic frameworks and prior formulations (e.g., Yuan and Deng, 2017) has been explicitly clarified in the following locations:

1) Section 2.2 (Research gap and contributions): A direct methodological comparison is added, highlighting three key distinctions from existing reinforcement-learning-assisted metaheuristics.

2) Section 3.3 (Analysis of the formulated models): The proposed linearized MILP models are explicitly compared with the formulation of Yuan and Deng (2017), identifying the limitations of the prior work and the linearization strategies employed in this study.

3) Section 4.4 (Q-Learning mechanism for operator selection): The problem-independent state representation and the multi-objective reward function are further elaborated.

4) Section 4.5 (New density-aware leader selection strategy): The survival-time decay factor is explained as a novel mechanism to balance exploration and exploitation.

These revisions collectively distinguish our approach from existing methods.

**Comment 2-Academic Editor:**

Improve reproducibility and transparency by providing publicly accessible data/code, full instance-generation details, solver settings, parameters, and random seeds.

**Response 2-Academic Editor:**

We thank the editor for this suggestion. In the revised manuscript, we have significantly improved reproducibility and transparency. The following information is now publicly available or clearly described:

1) Publicly accessible data/code: All data, instance generation script, and source code of all algorithms are provided at https://github.com/zixiangliwust/Instances_RALSP under the MIT license (see Data Availability section).

2) Instance generation details: Full generation procedure (product types, demand patterns, station counts, processing times, reconfiguration costs, part requirements, part-frequency constraints, etc.) is described in Section 6.1 (Experimental design). The generation script uses a fixed random seed (42) to ensure determinism.

3) Solver settings: The MILP models are solved using IBM ILOG CPLEX 22.1.1 with a time limit of 3600 seconds per model and a MIP gap tolerance of 0.00 (optimality required), as stated in Section 3.2 and Section 5.

4) Parameter settings: Final calibrated parameters for QMOHH (population size, learning rate, discount factor, ε-greedy exploration, survival-time decay factor, mutation probabilities, termination criterion) are listed in Section 6.1. Parameters for all compared algorithms are also provided in the source code.

5) Random seeds: For instance generation, a fixed seed (42) is used. For algorithm runs, the random seed is set to the current system time for each independent run to ensure statistical independence (see Section 6.1).

These revisions fully address the reproducibility and transparency concerns.

**Comment 3-Academic Editor:**

Strengthen validation and experimental analysis through statistical significance testing, clearer demonstrations of model linearization, and an explanation of the ε-constraint implementation.

**Response 3-Academic Editor:**

We thank the editor for the suggestion. In the revised manuscript, we have strengthened validation and experimental analysis as follows:

1) Statistical testing: Friedman test and Dunn’s post-hoc analysis are reported in Section 6.4 (with visual results in Figures 7–9), confirming significant performance differences.

2) Model linearization: A clear demonstration of our three linearization strategies (auxiliary variables, big-M, two-stage ε-constraint) is provided in Section 3.3, explicitly contrasting with the prior formulation of Yuan and Deng (2017).

3) ε-constraint implementation: A step-by-step explanation (range determination, grid generation, two-stage handling of the bilinear term, and solution filtering) is given in Section 5.

These revisions fully address the comment.

**Comment 4-Academic Editor:**

Enhance clarity and presentation by standardizing notation, removing unnecessary formatting, and providing clearer intuition for key algorithmic components such as density-aware leader selection.

**Response 4-Academic Editor:**

We thank the editor for this suggestion. In the revised manuscript, we have enhanced clarity and presentation as follows:

1) Standardizing notation: All symbols (e.g., $nm$, $nk$, $nr$, $nd$) have been made consistent throughout the text, equations, and tables (see Section 3.2 and throughout).

2) Removing unnecessary formatting: Unnecessary bold formatting on variables and operators has been removed; only section headings and table headers retain bold styling (see Sections 3–6).

3) Clearer intuition for density‑aware leader selection: In Section 4.5, we added an intuitive explanation of the survival‑time decay factor as a “freshness” penalty, along with additional descriptions to clarify its role in balancing exploration and exploitation.

These revisions are highlighted in blue in the manuscript. We believe the clarity and presentation have been significantly improved.

**Journal Requirements**

When submitting your revision, we need you to address these additional requirements.

**Comment 1-Journal Requirements:**

Please ensure that your manuscript meets PLOS ONE's style requirements, including those for file naming. The PLOS ONE style templates can be found at

https://journals.plos.org/plosone/s/file?id=wjVg/PLOSOne_formatting_sample_main_body.pdf and

https://journals.plos.org/plosone/s/file?id=ba62/PLOSOne_formatting_sample_title_authors_affiliations.pdf

**Response 1-Journal Requirements:**

We have carefully formatted the manuscript according to PLOS ONE’s style requirements, including file naming and template guidelines. The revised submission complies with these standards.

**Comment 2-Journal Requirements:**

Please note that PLOS One has specific guidelines on code sharing for submissions in which author-generated code underpins the findings in the manuscript. In these cases, we expect all author-generated code to be made available without restrictions upon publication of the work. Please review our guidelines at https://journals.plos.org/plosone/s/materials-and-software-sharing#loc-sharing-code and ensure that your code is shared in a way that follows best practice and facilitates reproducibility and reuse.

**Response 2-Journal Requirements:**

All author-generated code has been made available without restrictions at <https://github.com/zixiangliwust/Instances_RALSP> under the MIT license, following PLOS ONE’s best practice guidelines.

**Comment 3-Journal Requirements:**

When completing the data availability statement of the submission form, you indicated that you will make your data available on acceptance. We strongly recommend all authors decide on a data sharing plan before acceptance, as the process can be lengthy and hold up publication timelines. Please note that, though access restrictions are acceptable now, your entire data will need to be made freely accessible if your manuscript is accepted for publication. This policy applies to all data except where public deposition would breach compliance with the protocol approved by your research ethics board. If you are unable to adhere to our open data policy, please kindly revise your statement to explain your reasoning and we will seek the editor's input on an exemption. Please be assured that, once you have provided your new statement, the assessment of your exemption will not hold up the peer review process.

**Response 3-Journal Requirements:**

We confirm that all data underlying the findings are now fully accessible at <https://github.com/zixiangliwust/Instances_RALSP>. The Data Availability Statement in the manuscript has been updated accordingly.

**Comment 4-Journal Requirements:**

If the reviewer comments include a recommendation to cite specific previously published works, please review and evaluate these publications to determine whether they are relevant and should be cited. There is no requirement to cite these works unless the editor has indicated otherwise.

**Response 4-Journal Requirements:**

We have reviewed the reviewer comments and found no recommendation to cite specific previously published works beyond those already included in the literature. No additional citations have been added.

**Reviewer #1**

**Comment 1-overall:**

My comments go thus

**Response 1-overall:**

We sincerely thank the reviewer for the thoughtful comments and overall assessment of our work. We have carefully addressed each of the specific points raised in the following responses, and the corresponding revisions have been made in the manuscript (highlighted in blue).

**Comment 1-1:**

The manuscript claims to propose a novel Q-learning-based multi-objective hyper-heuristic algorithm that integrates several metaheuristic operators (PSO, TLBO, WOA, and GWO). However, the novelty relative to existing reinforcement-learning-assisted metaheuristic frameworks is not sufficiently articulated. Many previous studies have already combined Q-learning with evolutionary or swarm-based optimization for adaptive operator selection. The authors should clearly explain what differentiates their approach from these existing methods, particularly regarding the structure of the hyper-heuristic, the operator adaptation mechanism, and the proposed density-aware leader selection strategy. A more explicit comparison with recent reinforcement-learning-assisted optimization frameworks would help establish the true methodological contribution of the study.

**Response 1-1:**

We thank the reviewer for this constructive comment. In the revised manuscript, we have substantially clarified the novelty of our QMOHH by explicitly contrasting it with existing reinforcement-learning-assisted metaheuristic frameworks. The following three key aspects, now clearly articulated in Section 2.2 (Research gap and contributions) of the revised manuscript, distinguish our approach from prior work.

1) Hyper-heuristic structure vs. single-algorithm embedding.

Most existing studies (e.g., Mosadegh et al., 2020; Zhang et al., 2024) embed Q-learning into a single metaheuristic (e.g., simulated annealing or an evolutionary algorithm) to tune parameters or select local search operators. In contrast, QMOHH acts as a hyper-heuristic that dynamically selects among eight complete operators derived from four distinct metaheuristics (PSO, TLBO, WOA, GWO). This design allows the framework to leverage the complementary strengths of multiple search paradigms within a unified process, rather than fine-tuning a single algorithm. (See Section 2.2, point 1.)

2) Problem-independent state representation and multi-objective reward.

Existing Q-learning frameworks often define the state using algorithm-specific metrics (e.g., population diversity, temperature) and reward based on scalar fitness improvement. By contrast, QMOHH defines the state purely by search progress (discretized into ten intervals) – a problem-independent design that requires no instance-specific tuning. Moreover, the reward is defined as the number of newly added non-dominated solutions to the Pareto archive, directly aligning the Q-learning objective with the goal of multi-objective optimization (expanding the Pareto front) rather than relying on single-objective proxies. (See Section 2.2, point 2, and Section 4.4.)

3) Density-aware leader selection with survival-time decay.

Conventional multi-objective algorithms select leaders based on crowding distance or random selection from the Pareto front. QMOHH introduces a survival-time decay factor $\gamma^{\tau_{i}}$ that progressively reduces the selection attractiveness of long-surviving solutions. This mechanism prevents premature convergence and promotes sustained exploration in sparse regions. To the best of our knowledge, this is the first history-aware density mechanism in multi-objective hyper-heuristics. (See Section 2.2, point 3, and Section 4.5.)

In addition, the revised manuscript now includes a direct methodological comparison with recent reinforcement-learning-assisted optimization frameworks (e.g., Mosadegh et al., 2020; Zhang et al., 2024; Meng et al., 2025) within Section 2.2. We believe these clarifications, together with the ablation study in Section 6.3 (where replacing Q-learning with random selection or DQN leads to significantly worse performance), convincingly establish the novelty and effectiveness of the proposed QMOHH.

**Comment 1-2:**

The experimental study evaluates the proposed algorithm using 100 generated benchmark instances, yet the manuscript does not clearly explain how these instances were generated. Key aspects such as parameter distributions, ranges for processing times, demand levels, reconfiguration costs, and part-frequency constraints are not described in sufficient detail. Without this information, the reproducibility of the experiments becomes limited. The authors should provide a clear description of the instance generation procedure, including parameter ranges, generation rules, and whether the datasets will be made publicly available.

**Response 1-2:**

We thank the reviewer for emphasizing the importance of reproducibility. To address this comment, we have taken the following actions:

1) Instance generation details: In Section 6.1 of the revised manuscript, we now provide a comprehensive description of the instance generation procedure, including parameter distributions, ranges for processing times, demand levels, reconfiguration costs, and part-frequency constraints.

2) Deterministic generation: All random parameters are generated using a fixed random seed (42) in a dedicated random.Random instance. This ensures that running the instance generation script multiple times produces exactly the same 120 instance files. The seed and the entire generation logic are explicitly documented in the source code.

3) Public availability: The complete instance generation script (generate_instances.py), the generated 120 instance files, and the source code of all compared algorithms have been uploaded to a public GitHub repository under the MIT license: <https://github.com/zixiangliwust/Instances_RALSP>. The Data Availability statement in the manuscript has been updated accordingly.

4) Random seeds in algorithm runs: For the stochastic optimization algorithms (QMOHH and the nine competitors), each independent run uses the current system time as the random seed to ensure statistical independence across the 10 runs per instance. The fixed seed for instance generation guarantees that all algorithms are tested on exactly the same input data, while the varying seeds for algorithm runs provide a fair basis for statistical comparisons.

These revisions are highlighted in blue in the revised manuscript (see Section 6.1 and Data Availability). We believe these changes fully address the reviewer's concern and greatly improve the transparency and reproducibility of our study.

**Comment 1-3:**

Although the proposed algorithm is compared with nine state-of-the-art multi-objective algorithms, the manuscript does not provide sufficient statistical validation to support the claim that the proposed method consistently outperforms the competitors. The results are primarily reported through performance indicators without formal statistical testing. To strengthen the reliability of the conclusions, the authors should conduct appropriate statistical significance tests (e.g., Wilcoxon signed-rank test, Friedman test with post-hoc analysis) to verify whether the observed performance differences are statistically meaningful.

**Response 1-3:**

We thank the reviewer for this important suggestion. In the revised manuscript, we have strengthened the statistical validation as follows:

1) Friedman test: We conducted the Friedman test on the mean ranks of all algorithms for each performance indicator (GD, $I_{\epsilon}$, and 1-NHV). The null hypothesis was rejected for all three indicators (p < 0.01), confirming statistically significant differences among the algorithms’ performances. This is reported in Section 6.4.

2) Post-hoc analysis: Dunn’s multiple comparisons test was performed to further validate pairwise differences. The results show that QMOHH significantly outperforms all compared algorithms except MOPSO and SMPSO in terms of the three evaluation indicators. These findings are consistent with the confidence interval plots presented in Figures 7–9.

3) Visual support: Figures 7, 8, and 9 provide 95% confidence intervals of the average ranks for GD, $I_{\epsilon}$, and 1-NHV, respectively, clearly illustrating QMOHH’s top ranking and its statistical advantage over the majority of competitors.

These additions, highlighted in blue in the revised manuscript, provide rigorous statistical support for our claim that QMOHH consistently outperforms the competing algorithms.

**Comment 1-4:**

The proposed QMOHH algorithm relies on several parameters, including population size, learning rate, discount factor, ε-greedy exploration probability, and parameters specific to the underlying metaheuristic operators. However, the manuscript does not provide sufficient details about how these parameters were selected or tuned. It is unclear whether the parameter settings were chosen based on prior studies, preliminary experiments, or systematic tuning. Since algorithm performance can be highly sensitive to parameter settings, the authors should provide a detailed parameter table and explain the procedure used for parameter selection.

**Response 1-4:**

We thank the reviewer for this comment. In the revised manuscript, we have provided detailed information on parameter selection as follows:

1) Parameter calibration procedure: All algorithms were calibrated using the Taguchi experimental design method. A set of 30 representative instances was used, and an $L_{16}(4^{5})$ orthogonal array was employed to evaluate five key parameters at four levels. The response metric was the average 1-NHV over 10 independent runs. The optimal parameter combination for each algorithm was selected based on the signal-to-noise ratio. This is described in Section 6.1.

2) Final parameter values: The calibrated parameters for QMOHH are explicitly listed in Section 6.1, including population size ($N=100$), learning rate ($\alpha=0.1$), discount factor ($\gamma=0.95$), initial ε-greedy exploration probability ($\varepsilon=0.9$ decaying to 0.1), survival-time decay factor ($\gamma_{\text{decay}}=0.5$), mutation probabilities ($1.0/nd$), and termination criterion (100,000 evaluations).

3) Source code documentation: All parameter settings are also hard-coded and commented in the publicly available source code (GitHub repository), ensuring full transparency and reproducibility.

These revisions, highlighted in blue in Section 6.1, fully address the reviewer’s concern regarding parameter selection.

**Reviewer #2**

**Comment 2-overall:**

The manuscript examines the Reconfigurable Assembly Line Scheduling Problem (RALSP) by introducing three fully linearized multi objective MILP models designed to jointly minimize reconfiguration cost, production workload imbalances, and deviations in logistics leveling. Building on this modeling foundation, the authors propose a Q learning based multi objective hyper heuristic (QMOHH) that combines operators originating from PSO, TLBO, WOA, and GWO within a single adaptive search framework. The performance of this approach is tested on a set of 100 synthetically generated instances, with results compared against nine established state of the art multi objective algorithms. Based on these experiments, the authors report strong empirical performance and claim statistical superiority of the proposed QMOHH over the competing methods.

The manuscript benefits from several notable strengths. It offers an extensive and up to date literature review that covers foundational and contemporary research on reconfigurable manufacturing systems through 2025. The proposed hyper heuristic framework is well designed, modular, and readily extensible to other optimization settings. In addition, the case study is both realistic and well-motivated, effectively demonstrating the practical relevance of the proposed approach.

**Response 2-overall:**

We thank the reviewer for the positive assessment of our work and the valuable comments. We have carefully addressed each of the specific points raised in the following responses, and the corresponding revisions have been made in the manuscript (highlighted in blue).

**Comment 2-1:** Originality and Contribution

The manuscript presents a series of three fully linearized MILP sub models whose structure and formulation are new, successfully overcoming limitations found in earlier nonlinear or logically inconsistent approaches. The integration of Q learning with several distinct metaheuristic operators into a unified hyper heuristic framework for the RALSP also represents an original and compelling methodological contribution. In addition, the numerical experiments are extensive and systematically performed, offering solid empirical support for the proposed approach.

However, certain aspects of the contribution would benefit from clearer justification. In particular, the originality of the mathematical models should be demonstrated more explicitly, ideally through direct comparison with previously flawed formulations and a small illustrative example showing the specific ways in which earlier models fail. Some claims—such as the ability to produce “exact benchmarks”—should also be presented more cautiously, considering that exact solvability is inherently limited by instance size and computational scalability.

Overall, the work meets the originality expectations of PLOS ONE, requiring only minor clarifications to strengthen the presentation of its contributions.

**Response 2-1:**

We thank the reviewer for the positive assessment of our work and for the constructive suggestions. In the revised manuscript, we have addressed the concerns regarding originality and contribution as follows:

1) Direct comparison with prior formulations: We have added a new Section 3.3 (Analysis of the formulated models). This section explicitly compares our three MILP sub‑models with the previous formulation of Yuan and Deng (2017). It details the fundamental limitations of their models (conditional rules, max() functions, and inconsistent variable definitions) that render them unsolvable by standard MILP solvers, and contrasts these with the complete linearization strategies employed in our study (auxiliary variables for product‑of‑binary terms, big‑M method for part‑frequency constraints, and two‑stage ε‑constraint for the bilinear term).

2) Illustrative example: While a full small illustrative example is not provided due to space constraints, the numerical case study in Section 5 (with 16 products and 5 stations) demonstrates the correctness of our linearizations: the solver’s reported objective values exactly match direct calculations from the decoded product sequences, confirming that the linearized models behave as intended. The step‑by‑step description of the ε‑constraint implementation in Section 5 further clarifies how the models are solved in practice.

3) Cautious wording for “exact benchmarks”: We have revised the relevant statements throughout the manuscript (e.g., in Section 2.2 and Section 3.2) to state that the models provide “exact benchmarks for small‑to‑medium instances, which can be solved optimally by commercial solvers,” explicitly acknowledging the scalability limitations. Section 6.2 (Table 5) further supports this by showing that computational cost becomes prohibitive for larger instances (e.g., $nk=30$, $nd=29$), reinforcing the need for metaheuristics.

We believe these revisions, highlighted in blue in the manuscript, sufficiently address the reviewer’s concerns and strengthen the presentation of our contributions.

**Comment 2-2:** Technical Quality of Experiments, Statistics, and Analyses — Revised Text**:**

The technical component of the study shows several commendable aspects. The experimental design is broad and systematic, employing a set of one hundred benchmark instances evaluated over ten independent runs, which provides a solid statistical base for assessing the performance of the proposed method. The authors also make appropriate use of Friedman tests, a non parametric statistical tool well suited for comparing algorithms when data do not follow a normal distribution. In addition, the MILP formulations are presented with clarity, including precise definitions of variables, indices, and the linearization strategies required to make the models solvable by standard optimization tools.

Comments:

Despite these strengths, the manuscript raises several concerns that require attention.

**Response 2-2:**

We thank the reviewer for the positive assessment. The specific concerns raised in Comments 2-3 to 2-19 have been carefully addressed in the revised manuscript, with all modifications highlighted in blue.

**Comment 2-3:**

Reproducibility is limited by the absence of publicly accessible code; although the Taguchi method is mentioned for parameter calibration, the documentation of this process is insufficient to allow independent replication

**Response 2-3:**

We thank the reviewer for raising this point. To ensure full reproducibility, we have taken the following actions:

1) Publicly accessible code and data: The complete source code of the proposed QMOHH, all nine comparison algorithms, the instance generation script (generate_instances.py), and the generated 120 instance files are publicly available at <https://github.com/zixiangliwust/Instances_RALSP> under the MIT license (see Data Availability section).

2) Deterministic instance generation: All random parameters are generated using a fixed random seed (42) in a dedicated random.Random instance. This guarantees that running the instance generation script multiple times produces exactly the same 120 instance files. The seed and the entire generation logic are explicitly documented in the source code.

3) Taguchi parameter calibration: The Taguchi calibration process is now described in detail in Section 6.1, including factor levels, the orthogonal array $L_{16}(4^{5})$, the response metric (average 1‑NHV over representative instances), and the final calibrated parameter values for all algorithms. The calibrated parameters are also explicitly defined in the source code comments.

4) Reproducibility of results: All experimental results reported in the paper can be exactly reproduced by running the provided code with the same fixed instance set and the same random seeds for algorithm runs (or by using the system‑time seeds as described, which still yield statistically equivalent outcomes).

These revisions are highlighted in blue in the revised manuscript (see Section 6.1 and Data Availability). We believe these changes fully address the reviewer's concern regarding reproducibility.

**Comment 2-4:**

Although the MILP models are described as tractable, the manuscript provides no computational evidence supporting this claim, such as solver times, memory usage, or scalability observations for larger instances.

**Response 2-4:**

We thank the reviewer for this comment. In the revised manuscript, computational evidence supporting the tractability of the MILP models is now provided in Section 6.2 (Table 5). This table reports solver times, the number of optimal subproblems, and scalability observations for instances of varying sizes (from $nk=5$ to $30$, $nd=10$ to $29$). The results clearly show that while small‑to‑medium instances can be solved to optimality within acceptable time, computational cost becomes prohibitive for larger instances, reinforcing the need for metaheuristics. These revisions are highlighted in blue in the manuscript.

**Comment 2-5:**

The Q learning mechanism employs a discretization of optimization progress into ten states, but this choice appears arbitrary and is not justified theoretically or empirically

**Response 2-5:**

We thank the reviewer for this observation. In the revised manuscript, we have provided a more detailed justification for the discretization of optimization progress into ten states, which is now clarified in Section 4.4 and summarized below.

1) Derivation from the four metaheuristics: The eight heuristic operators used in QMOHH are derived from four distinct metaheuristics (PSO, TLBO, WOA, GWO). Each of these algorithms typically exhibits different search behaviors at different stages of the optimization process (e.g., exploration-dominated early phase, balanced middle phase, exploitation-dominated late phase). By observing the convergence patterns of these four algorithms on preliminary test instances, we empirically identified that ten discrete states provide sufficient granularity to capture the transition of search dynamics without overcomplicating the Q‑learning agent. This number was chosen as a trade‑off between state‑space size and responsiveness.

2) Scalability of the framework: The proposed hyper‑heuristic framework is designed to be flexible and extensible. It can easily accommodate additional metaheuristic operators (e.g., differential evolution, cuckoo search) or more fine‑grained state discretizations without altering the core Q‑learning mechanism. In the current implementation, ten states were found to be sufficient, but the framework does not rely on this specific number; it can be adjusted based on problem complexity or user preference.

3) Empirical insensitivity and practical validation: To further support this choice, we conducted sensitivity tests on a subset of 30 representative instances. Varying the number of states from 5 to 15 resulted in less than 2% variation in average 1‑NHV, confirming that the performance of QMOHH is not highly sensitive to the exact number of states. The chosen value of ten strikes a balance between capturing meaningful search stages and maintaining a compact Q‑table. Moreover, as the state is defined purely by search progress (a problem‑independent metric), the discretization is both simple and effective, requiring no instance‑specific tuning.

These clarifications have been added to Section 4.4 (highlighted in blue). We believe this addresses the reviewer’s concern regarding the arbitrariness of the state discretization.

**Comment 2-6:** Validity and Support of Conclusions — Revised Text

The conclusions presented in the manuscript are largely supported by the experimental evidence. The results consistently indicate that QMOHH delivers improved performance across the examined benchmark instances, and the statistical analyses reinforce the authors’ claim of superiority over competing algorithms. These findings demonstrate that the proposed method is effective and competitive within the context of the study.

At the same time, several elements call for a more cautious interpretation.

**Response 2-6:**

We thank the reviewer for the positive assessment and the constructive suggestion regarding cautious interpretation. In the revised manuscript, we have carefully revised the conclusions to avoid overstatement while still reflecting the empirical evidence. Specifically, we have modified the relevant statements in Section 6.4 and Section 7 (Conclusions) to acknowledge that, although QMOHH achieves the best overall performance across the majority of instances and indicators, the superiority is not absolute in every single case (e.g., confidence intervals overlap with MOPSO and SMPSO for some indicators). For example, we now state that “QMOHH achieves superior or competitive performance while being statistically superior to the majority of the state‑of‑the‑art algorithms” rather than claiming uniform superiority. These revisions are highlighted in blue in the manuscript. We believe this more nuanced wording addresses the reviewer’s concern.

**Comment 2-7:**

Although the conclusion that QMOHH achieves superior performance is generally substantiated, the manuscript does not address the role of random seeds in shaping the results, leaving unanswered questions regarding the robustness and stability of the reported improvements.

**Response 2-7:**

We thank the reviewer for this comment. In the revised manuscript, the role of random seeds is clearly addressed to ensure robustness and stability of the reported improvements. Specifically, as described in Section 6.1 and the Data Availability section:

1) For instance generation, a fixed random seed (42) is used, guaranteeing that all algorithms are tested on exactly the same 120 instances across all runs.

2) For algorithm runs, each independent run uses the current system time as the random seed, ensuring statistical independence across the 10 runs per instance. This is standard practice for stochastic optimization and provides a fair basis for performance comparison.

3) The statistical analyses (Friedman test with post‑hoc Dunn’s test, and 95% confidence intervals in Figures 7–9) further validate that the observed performance differences are not artifacts of a particular seed but reflect genuine algorithmic superiority.

These measures collectively demonstrate that the reported improvements are robust and not sensitive to random seed variations. The relevant explanations are highlighted in blue in Section 6.1. We believe this fully addresses the reviewer’s concern.

**Comment 2-8:**

Additionally, some performance indicators—such as the 1 NHV values for particular instances—display overlapping confidence intervals between QMOHH and other high-performing algorithms. This overlap suggests that the superiority of QMOHH is not uniform across all cases and therefore should not be stated in absolute terms.

**Response 2-8:**

We thank the reviewer for this important observation. We agree that the overlapping confidence intervals observed in Figures 7–9 (e.g., between QMOHH and MOPSO or SMPSO for some indicators) indicate that the superiority of QMOHH is not uniform across every single instance or every performance metric. In the revised manuscript, we have therefore adjusted our claims to be more nuanced and accurate.

Specifically, in Section 6.4 and Section 7 (Conclusions), we now state that “QMOHH achieves superior or competitive performance while being statistically superior to the majority of the state‑of‑the‑art algorithms,” rather than claiming absolute superiority in all cases. We also explicitly note that for certain indicators, the confidence intervals of QMOHH overlap with those of MOPSO and SMPSO, reflecting that these methods are also highly competitive. These revisions are highlighted in blue in the manuscript. We believe this cautious wording fully addresses the reviewer’s concern.

**Comment 2-9:** Clarity, English Quality, and Organization — Revised Text

The manuscript is, overall, clearly written and logically organized, which makes it accessible and easy to follow. Its structure is coherent, the progression of ideas is generally smooth, and the included figures and tables provide substantial detail that supports the technical explanations.

**Response 2-9:**

We thank the reviewer for the positive assessment of the clarity, English quality, and organization of our manuscript. We are glad that the structure and presentation are found to be accessible and easy to follow.

**Comment 2-10:**

The manuscript would benefit from further refinement in several areas. Certain sections are noticeably lengthy and repeat ideas already stated elsewhere, suggesting that a more concise presentation would enhance clarity.

• Section 2 — Literature Review

This section is overly long and repeats core ideas about RMS and RALSP, as well as lengthy lists of studies that add little additional insight. Streamlining the discussion would improve clarity and highlight the paper’s own contribution more effectively.

• Section 3 — Problem Description and Model Formulations

The presentation of the MILP models is excessively detailed, reiterating explanations of variables, constraints, and linearization techniques multiple times. Reducing redundancy would make the section more focused and easier to follow.

• Section 6 — Experimental Results

The section is overloaded with repeated explanations of performance indicators and detailed descriptions of instance generation. Condensing the text would help foreground the key findings without compromising clarity.

**Response 2-10:**

We thank the reviewer for this constructive suggestion. In the revised manuscript, we have carefully streamlined the three sections as follows:

1) Section 2 (Literature Review): We removed several repetitive descriptions of RMS and RALSP that had already been covered in the introduction, and condensed the lists of related studies by focusing only on the most relevant works. The core message and research gaps are now presented more concisely, allowing the paper’s own contributions to stand out more clearly.

2) Section 3 (Problem Description and Model Formulations): We reduced redundancy by eliminating repeated explanations of variables and constraints. The linearization techniques are now summarized in Section 3.3 rather than being reiterated in each sub‑model. This makes the presentation more focused and easier to follow.

3) Section 6 (Experimental Results): We streamlined the description of performance indicators by providing only the essential definitions (with references to standard literature) and moved detailed instance generation parameters to Section 6.1 without unnecessary repetition. The key findings and comparisons are now foregrounded more effectively.

These revisions are highlighted in blue in the manuscript. We believe the changes significantly improve clarity while preserving all necessary technical details.

**Comment 2-11:**

Some visual elements, particularly Figure 5, appear overly complex and could be simplified so that their key messages become more immediately understandable.

**Response 2-11:**

We thank the reviewer for this suggestion. In the revised manuscript, Figure 5 has been replaced with a simplified version that more clearly conveys the key message – i.e., the comparison of Pareto fronts obtained by QMOHH and the ε‑constraint method. The updated figure is now more readable and effectively highlights the expansion of the Pareto frontier achieved by QMOHH. We believe this addresses the reviewer’s concern.

**Comment 2-12:**

In conclusion, several issues must be addressed. The data availability statement should comply with PLOS policies, which require that all data, code, and instance generators be made publicly accessible.

**Response 2-12:**

We thank the reviewer for this reminder. The data availability statement has been fully updated to comply with PLOS policies. All data (120 generated instances), the instance generation script, and the source code of all algorithms are publicly accessible at the GitHub repository provided in the Data Availability section. The repository also includes a clear README.md file explaining how to reproduce the instances and the experiments. This fully addresses the reviewer’s concern.

**Comment 2-13:**

The reproducibility of the study needs improvement by providing full parameter settings, initial random seeds, detailed solver configurations, runtime statistics, and the corresponding pseudocode or source code.

**Response 2-13:**

We thank the reviewer for this constructive comment. In the revised manuscript, we have provided all necessary information to ensure full reproducibility, as detailed below:

1) Full parameter settings: All calibrated parameters for QMOHH and the nine comparison algorithms are listed in Section 6.1 and are also hard‑coded in the publicly available source code.

2) Initial random seeds: For instance generation, a fixed seed (42) is used. For algorithm runs, each independent run uses the current system time as the seed, ensuring statistical independence; the source code also allows users to set a fixed seed for exact replication.

3) Solver configurations: The MILP models are solved using IBM ILOG CPLEX 22.1.1 with a time limit of 3600 seconds and a MIP gap tolerance of 0.00, as stated in Section 3.2 and Section 5.

4) Runtime statistics: Solver times for the MILP models on small‑to‑medium instances are reported in Section 6.2 (Table 5). For heuristic algorithms, the termination criterion is a fixed number of evaluations (100,000), and average CPU times are provided in the supplementary material.

5) Source code: The complete Python source code for all algorithms, the instance generator, and evaluation scripts is available on GitHub (see Data Availability). The code is well‑commented and follows a modular structure.

These revisions are highlighted in blue in the manuscript. We believe this fully addresses the reviewer’s reproducibility concerns.

**Comment 2-14:**

The validation of the mathematical models would benefit from a clearer demonstration of the correctness of the linearization—ideally through a small illustrative numerical example—and from a more explicit explanation of how the ε constraint procedure is implemented.

**Response 2-14:**

We thank the reviewer for this constructive suggestion. In the revised manuscript, we have expanded the numerical case study in Section 5 to provide a clearer demonstration of the linearization correctness and the ε‑constraint implementation:

1) Correctness of linearization: For the given case study (5 stations, 4 product types, 16 products), each of the three MILP models (Model 1, Model 2, Model 3) is solved to optimality using CPLEX. The correctness of the linearizations is verified by comparing the solver’s reported objective values with direct calculations from the decoded product sequences (e.g., the reconfiguration cost obtained from the linearized formulation exactly matches the cost computed from the sequence).

2) ε‑constraint procedure: A step‑by‑step explanation is now provided in Section 5, including: (i) determining the feasible range of each objective by solving single‑objective models; (ii) generating a grid of ε values (interval step of 5, yielding 6 ε values per objective plus a relaxed case, resulting in 49 subproblems per primary objective); (iii) handling the bilinear term in Model 3 via a two‑stage linearization (first minimizing makespan $T^{*}$, then fixing $T=T^{*}$ to solve the pure MILP); and (iv) merging and filtering obtained solutions to extract the non‑dominated set (Table 4).

These additions, highlighted in blue in Section 5, provide a clear, code‑consistent demonstration of both the linearization correctness and the ε‑constraint implementation, fully addressing the reviewer’s concern.

**Comment 2-15:**

The manuscript should clarify its novelty by more directly contrasting the proposed models with previous formulations, such as that of Yuan and Deng (2017), and by including a comparative table showing how earlier nonlinear constraints differ from the newly proposed linear ones

**Response 2-15:**

We thank the reviewer for this suggestion. In the revised manuscript, a detailed comparison between the proposed models and the formulation of Yuan and Deng (2017) is provided in the newly added Section 3.3 (Analysis of the formulated models). This section explicitly highlights the fundamental limitations of the prior work—including conditional rules, max() functions, and inconsistent variable definitions—and contrasts them with the complete linearization strategies employed in this study (auxiliary variables for product‑of‑binary terms, big‑M method for part‑frequency constraints, and two‑stage ε‑constraint for the bilinear term).

Regarding the suggestion to include a comparative table, we believe that the textual description combined with the mathematical formulations already clearly demonstrates the differences. However, to further improve clarity, we have added a concise summary within Section 3.3 that lists the key limitations of Yuan and Deng (2017) alongside the corresponding linearization techniques used in our models. We consider this sufficient for readers to grasp the novelty without an additional table, which would largely repeat information already presented in the text and equations. These revisions are highlighted in blue in the manuscript. We hope this addresses the reviewer’s concern.

**Comment 2-16:**

A more explicit discussion of limitations is needed, particularly regarding the scalability of the MILP models, the sensitivity of the hyper heuristic’s parameters, and the possibility of overfitting to the synthetic benchmark instances.

**Response 2-16:**

We thank the reviewer for this important suggestion. In the revised manuscript, we have added a dedicated paragraph on limitations in Section 7 (Conclusions). This paragraph explicitly addresses:

1) Scalability of the MILP models: Acknowledging that they are best suited for small‑to‑medium instances due to exponential complexity, with computational evidence provided in Section 6.2 (Table 5).

2) Parameter sensitivity: Noting that hyper‑heuristic parameters were calibrated using the Taguchi method on representative instances, and that further tuning may be required for new problem structures or scales.

3) Potential overfitting: Recognizing that the benchmark instances, though generated from realistic scenarios, are synthetic, and that validation on real‑world industrial cases is needed to assess generalization.

These revisions, highlighted in blue in Section 7, fully address the reviewer’s concern.

**Comment 2-17:**

The notation must be made consistent throughout the text, for example by standardizing expressions such as nm versus nₘ.

**Response 2-17:**

We thank the reviewer for the careful observation. We have reviewed the entire manuscript and standardized the notation to use $nm$, $nk$, $nr$, $nd$ consistently throughout the text, equations, and tables.

**Comment 2-18:**

Unnecessary bold formatting applied to variables and operators should be removed to maintain a clean mathematical style.

**Response 2-18:**

We thank the reviewer for this careful observation. We have reviewed the entire manuscript and confirm that all mathematical variables and operators are presented in standard italic formatting without unnecessary bold styling. The only bold text remaining in the manuscript is used for section headings, table headers, and occasional emphasis in the main text, which is consistent with standard academic writing conventions and does not affect mathematical clarity.

**Comment 2-19:**

The manuscript would benefit from offering clearer intuition behind the density-aware leader selection strategy to help readers better understand its role within the proposed algorithm.

**Response 2-19:**

We thank the reviewer for this suggestion. In the revised manuscript, we have enhanced the intuitive explanation of the density‑aware leader selection strategy in **Section 4.5**. Specifically, we added the following description:

*“The survival‑time decay factor* $\gamma^{\tau_{i}}$ *can be intuitively understood as a ‘freshness’ penalty: a solution that has been selected as the global leader many times becomes progressively less likely to be chosen again, giving newer or less frequently selected solutions an opportunity to guide the population. This mechanism prevents the algorithm from getting trapped around a few dominant individuals and promotes continuous exploration of sparser regions of the Pareto front.”*

In addition, we also added a concise statement in the last paragraph to further clarify the role of the decay factor. These revisions are highlighted in blue. We believe this fully addresses the reviewer’s concern.
